# Infections with Tickborne Pathogens after Tick Bite, Austria, 2015–2018

**DOI:** 10.3201/eid2704.203366

**Published:** 2021-04

**Authors:** Mateusz Markowicz, Anna-Margarita Schötta, Dieter Höss, Michael Kundi, Christina Schray, Hannes Stockinger, Gerold Stanek

**Affiliations:** Medical University of Vienna, Vienna, Austria (M. Markowicz, A.-M. Schötta, M. Kundi, C. Schray, H. Stockinger, G. Stanek);; Private Medical Office, Thiersee, Austria (D. Höss)

**Keywords:** Lyme borreliosis, Lyme disease, Borrelia burgdorferi sensu lato, bacteria, rickettsia, Anaplasma phagocytophilum, Candidatus Neoehrlichia mikurensis, Borrelia miyamotoi, ticks, tick bite, tick-borne pathogens, Ixodes ricinus, vector-borne infections, zoonoses, Austria

## Abstract

The aim of this prospective study was to assess the risk for tickborne infections after a tick bite. A total of 489 persons bitten by 1,295 ticks were assessed for occurrence of infections with *Borrelia burgdorferi* sensu lato, *Anaplasma phagocytophilum*, *Rickettsia* spp., *Babesia* spp., *Candidatus* Neoehrlichia mikurensis, and relapsing fever borreliae. *B. burgdorferi* s.l. infection was found in 25 (5.1%) participants, of whom 15 had erythema migrans. Eleven (2.3%) participants were positive by PCR for *Candidatus* N. mikurensis. One asymptomatic participant infected with *B. miyamotoi* was identified. Full engorgement of the tick (odds ratio 9.52) and confirmation of *B. burgdorferi* s.l. in the tick by PCR (odds ratio 4.39) increased the risk for infection. *Rickettsia helvetica* was highly abundant in ticks but not pathogenic to humans. Knowledge about the outcome of tick bites is crucial because infections with emerging pathogens might be underestimated because of limited laboratory facilities.

## Abstract 2

Knowledge about outcomes of tick bites is crucial because infections with emerging pathogens might be underestimated.

Ticks are vectors for a variety of tickborne pathogens that cause human disease (*1*). The diversity of tickborne pathogens has increased extensively in recent years, supported by progress in the molecular identification of microorganisms (*2*). Clinical studies on the health-related impact of many emerging tickborne pathogens are scarce and information on the epidemiology is limited.

We undertook a comprehensive observational study in Austria to assess the incidence of recognized tickborne infections by applying clinical, serologic, and microbiological endpoints. We conducted a detailed risk analysis of contracting Lyme borreliosis. Our objective was to investigate whether variables such as confirmation of *Borrelia burgdorferi* sensu lato in ticks, duration of tick attachment, engorgement of ticks, and number of simultaneous tick bites have an impact on the risk for infection. Furthermore, we wanted to know whether the localization of a given tick bite and any previous contact with *B*. *burgdorferi* s.l. can affect this risk.

## Methods

Participants were enrolled prospectively during 2015–2018 at 2 centers in Austria (Vienna and Thiersee). The invitation to participate was announced in the local media. The analysis focused on infections with tickborne pathogens including *B. burgdorferi* s.l., *Anaplasma phagocytophilum*, *Rickettsia* spp., *Babesia* spp., *Candidatus* Neoehrlichia mikurensis, and relapsing fever borreliae. The study was approved by the ethics committee of the Medical University of Vienna (1064/2015) and of the Medical University of Innsbruck (AN2016-0043-359/4.16). Participants provided written informed consent.

### Inclusion/Exclusion Criteria

Inclusion criteria were a minimum age of 18 years and the availability of the particular tick for testing. Persons bitten >7 days before assessment were excluded.

### Questionnaire

A standardized questionnaire was used to collect information concerning tick bite location, history of erythema migrans, antimicrobial drug treatment within 4 weeks before the tick bite, estimated duration of tick attachment, number of ticks removed, and possible geographic region of tick attack. The feeding duration of the tick was reported in days by the difference of the estimated date of the tick bite and the date of tick removal.

### Outcome Definition

Serologic testing and PCR for blood were conducted during the first week after the removal of the tick, with a follow-up scheduled 6 weeks thereafter. We defined infection as >1 of the following: occurrence of erythema migrans diagnosed by a medical professional (M.M. or D.H.), increase in *Borrelia*-specific antibodies in follow-up samples, and presence of the microorganism determined by PCR in the initial or follow-up blood samples.

### Laboratory Analyses

Laboratory analyses were conducted at the Institute of Hygiene and Applied Immunology in Vienna. An experienced technician (A.-M.S.) identified ticks morphologically. If identification was inconclusive, we used molecular methods. Ten percent of the randomly selected *Ixodes ricinus* ticks underwent molecular identification to confirm the identification procedure. We documented the developmental stage of the ticks and recorded engorgement levels as not engorged, partially engorged, or fully engorged.

We extracted DNA from the ticks as described (*2*). Molecular identification of ticks was conducted by using the mitochondrial 16S rRNA gene (*3*), 12S rDNA gene (*4*), internal transcribed spacer 2 region (*5*), or cytochrome c oxidase subunit 1 gene (*6*). PCR products were purified and sent to Microsynth (https://www.microsynth.at) for bidirectional sequencing.

Molecular detection of *B. burgdorferi* s.l.; *Rickettsia* spp.; *Anaplasma*/*Ehrlichia* spp., including *Candidatus* N. mikurensis, *Babesia* spp., and *Coxiella burnetti*; was performed by using reverse line blot (RLB) hybridization (*2*). Sequencing was conducted if RLB failed to yield a species-specific signal. When *Rickettsia* spp. could not be identified by sequencing the 23S–5S intergenic spacer region used for RLB (*7*), we conducted additional PCRs specific for the *gltA* gene (*8*,*9*). We used real-time PCRs to detect *B. miyamotoi* (*10*) and, in addition to RLB hybridization, for *Candidatus* N. mikurensis (*11*).

### Molecular Analysis of Blood

We screened extracted DNA from blood containing EDTA for tickborne pathogens by using real-time PCR. The pathogens screened were *B. burgdorferi* s.l. (*12*), *Rickettsia* spp. (*12*), relapsing fever borreliae (*12*), *A. phagocytophilum* (*13*), *B. miyamotoi* (*10*), and *Candidatus* N. mikurensis (*11*).

### Serologic Testing

We assessed infections with *B. burgdorferi* s.l. by comparing ELISA values for IgM and IgG at the first and the follow-up tests. The increase in antibody levels was observed when the first sample yielded a negative result by using the cutoff value provided by the manufacturer and the result was positive in the follow-up sample. For specimens with a value above the cutoff value in the initial sample, we defined the infection as a 25% increase. Positive and borderline ELISA results were confirmed by using immunoblot (Anti-Borrelia-EUROLINE-RN-AT; Euroimmun, https://www.euroimmun.com).

During this study, a change of test systems was necessary because of withdrawal of systems from the market. A *Borrelia* ELISA (Medac, https://international.medac.de) was used until the end of May 2018, followed by Anti-Borrelia-plusVlsE-ELISA (Euroimmun) after June 2018. In the instance that the first and the follow-up serum samples were analyzed by different ELISAs, we used a paired sample for retesting with the new ELISA.

We performed serologic testing for other tickborne pathogens by applying the following commercial tests: *A. phagocytophilum* and *Rickettsia* IgG immunofluorescence assays (Focus Diagnostics, https://www.focusdx.com) and the Weil-Felix agglutination assay (DiaMondiaL, https://www.diamondial.com) as an additional serologic test able to detect infections with *Rickettsia* spp. Infections were defined as a 4-fold change in the titer.

### Determination of Sample Size

Human infection rates for *B. burgdorferi* s.l. after tick bites have been reported to be 2%–5% (*14*,*15*). Because high endemicity can be assumed for the covered regions, we determined the sample size on the basis of an upper limit of 5%. To have a power of 80% to detect an effect associated with an odds ratio (OR) >2, and considering covariates with a combined R^2^ of 25%, a total of 411 participants were considered necessary to provide statistical significance at the (2-sided) 5% level.

### Statistical Analysis

Because many participants had >1 tick on >1 occasion, we considered only ticks brought at the time of infection for infected persons. For noninfected persons who had >1 visit, 1 visit was chosen randomly, and the tick removed on that occasion was used for analysis. Similarly, if several ticks were available for the visit, 1 tick was randomly selected unless 1 of them was infected.

Preliminary comparisons of *Borrelia*-infected and noninfected participants were performed by using the Mann-Whitney test for metric data. We used the Fisher exact probability test for dichotomous data and the Fisher-Freeman-Halton test for categorical data. These data are reported as mean ± SD and median (interquartile range) with absolute and percent frequencies. Multiple logistic regression analysis was conducted to assess the risk for infection associated with attributes of the ticks, taking the age and sex of the participants into account. Seven persons did not complete follow-up testing and were excluded from the analyses. No imputation for missing data was applied. All analyses were performed by using Stata 13.1 (StataCorp LLC, https://www.stata.com).

## Results

### Study Population

A total of 489 participants were included in the study, of whom 7 were unavailable for follow-up. The number of ticks removed by the participants was 1,295. The final total of 482 study participants (255 women and 227 men) had a mean age of 49 years (range 19–83 years) and had been bitten by 1,279 ticks. A total of 433 (89.8%) participants were enrolled in Vienna and 49 (10.2%) were enrolled in Tyrol. At baseline, 120 (24.9%) participants were seropositive for *Borrelia* antibodies, 39 (8.1%) for *A. phagocytophilum* antibodies, and 13 (2.7%) for *Rickettsia* spp. antibodies. The mean time interval between the baseline and the follow-up test was 47 days (range 21–147 days).

### Ticks Obtained from Participants

A total of 96% of the tick bites occurred in Austria. Most ticks were removed during the months of June (338, 26.4%) and May (303, 23.7%), followed by July (227, 17.7%) and August (115, 9.0%).

Of the 1,279 ticks, 1,277 (99.8%) were *I. ricinus*. The 2 remaining ticks were *H. concinna* and a nymphal *Haemaphysalis* sp. tick imported from Cambodia. The most common developmental stage was the nymphal stage (922 ticks, 72.1%) followed by larvae (241 ticks, 18.8%), and adults (112 ticks [103 females and 9 males], 8.8%). For 4 ticks (0.3%), it was not possible to identify the developmental stage, but *I. ricinus* was confirmed by PCR.

**Figure 1:**
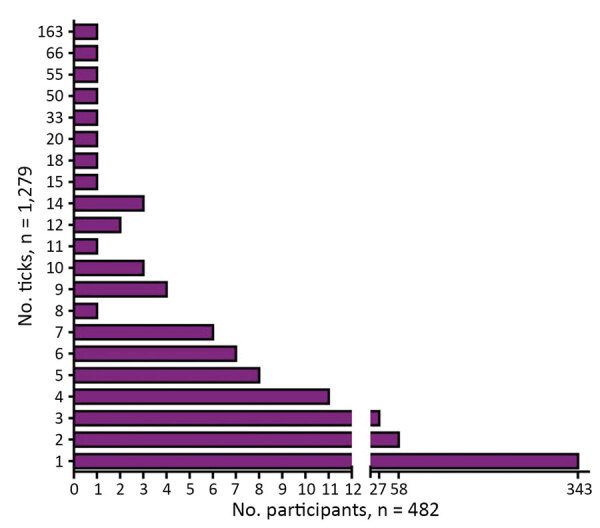
Number of ticks per study participant in study of infections with tickborne pathogens after tick bite, Austria, 2015–2018.

**Figure 2:**
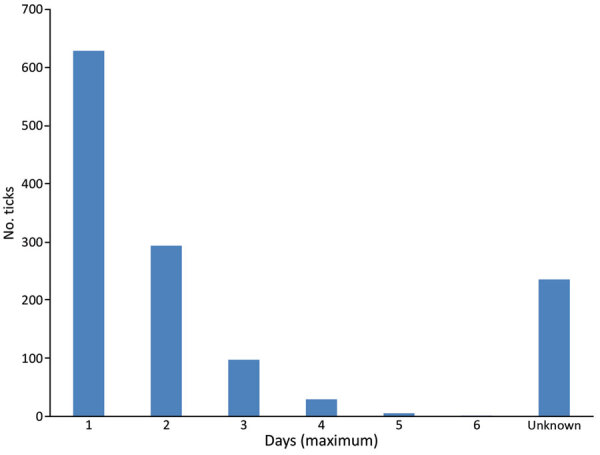
Estimated duration of tick attachment (n = 1,279) for infections with tickborne pathogens after tick bite, Austria, 2015–2018.

We compiled an overview of tick collection (Figure 1). Of the 482 participants, 139 persons collected >1 tick. The highest number of ticks per person was 163. Nearly half of the ticks were removed on the first day (629, 49.2%) (Figure 2).

### Molecular Screening of Ticks

*B. burgdorferi* s.l.was detected in 15.2% (194/1,279) of all ticks. The most common genospecies was *B. afzelii* in 66.5% (129/194), followed by *B. garinii*/*B. bavariensis* in 16.5% (32 ticks), *B. burgdorferi* sensu stricto in 7.7% (15 ticks), and other *Borrelia* spp. in 11.3% (22 ticks). Co-infections with >1 genospecies were detected in 4 ticks.

*Rickettsia* spp. was the second most frequent organism with 9.4% (120/1,279), and *R. helvetica* represented 86.7% (104/120) of all *Rickettsia*-positive ticks, followed by *R. monacensis* in 8 ticks (6.7%). Eight *Rickettsia*-positive samples yielded only genus-specific signals on the RLBs. Presence of *Candidatus* R. mendelii was confirmed by sequencing 4 of these ticks. Two were new species according to phylogenetic guidelines (*16*), of which 1 belonged to the spotted fever group *Rickettsiae* (*17*). For the remaining 2 *Rickettsia*-positive ticks, the species could not be identified. We provide an overview of the tickborne pathogens detected in the different life stages of the ticks (Table 1).

**Table 1:** Tickborne pathogens detected in different life stages of ticks after tick bite, Austria, 2015–2018

Pathogen or tick	Tick life stage					
	Adult females	Adult males	Nymphs	Larvae	Not identified	Total
*Borrelia burgdorferi* sensu lato	29	3	159	1	2	194
*B. afzelii*	10	1	115	1	2	132
*B. garinii/B. bavariensis*	7	1	24	0	0	32
*B. burgdorferi* sensu stricto	3	0	12	0	0	15
*B. valaisiana*	5	1	8	0	0	14
*B. lusitaniae*	1	0	1	0	0	2
*B. spielmanii*	3	0	3	0	0	6
Co-infections	0	0	4	0	0	4
*Rickettsia* spp.	14	0	69	37	0	120
* R. helvetica*	12	0	56	36	0	104
* R. monacensis*	1	0	6	1	0	8
*Candidatus* R. mendelii	1	0	3	0	0	4
New endosymbiont	0	0	1	0	0	1
*Candidatus* R. thierseensis	0	0	1	0	0	1
Not identified	0	0	2	0	0	2
*Anaplasmataceae*						
*Candidatus* Neoehrlichia mikurensis	5	1	46	1	1	54
* Anaplasma phagocytophilum*	1	0	29	0	0	30
*Babesia* spp.	3	0	20	5	0	28
* B. microti*	3	0	18	0	0	21
* B. divergens*	0	0	1	0	0	1
* B.. venatorum*	0	0	1	5	0	6
Relapsing fever borreliae						
* B. miyamotoi*	1	0	20	2	1	24

Of the 1,279 ticks included in the study, 380 (29.7%) harbored >1 tickborne pathogen. Dual infections with organisms of different genera occurred in 48 ticks (3.8%). Seven ticks (0.6%) harbored 3 different genera.

### Human Infection

*Borrelia* infection was found in 25 (5.1%) participants. Fifteen patients had erythema migrans, of whom 9 also showed an increase in *Borrelia*-specific antibodies in the follow-up sample. All instances of erythema migrans except 1 were localized at the site of the tick bite. Moreover, in 10 persons, evidence of *Borrelia* infection was found by serologic testing, and these persons did not have erythema migrans or any other symptoms. Demonstration of *B. burgdoferi* s.l. by PCR in the blood was successful in only 1 participant who had erythema migrans in an early stage. Infection with *B. burgdorferi* s.l. occurred twice in 2 participants. One woman had an erythema migrans twice within 4 months. Another woman had an asymptomatic infection, followed by erythema migrans 3 weeks later. She had been bitten by 11 ticks and showed seroconversion. Thereafter, she had another tick bite, which caused also erythema migrans around the bite. Antimicrobial drugs were given to patients who had erythema migrans but not to those who had asymptomatic infections.

With regard to other infections, 11 (2.3%) participants were positive for *Candidatus* N. mikurensis. These participants reported no symptoms. For 3 participants, the presence of *Candidatus* N. mikurensis was identified at the first visit, as well as at the follow-up tests. The time intervals between the examinations for these 3 participants were 41, 44, and 86 days. One study participant was positive for *B. miyamotoi* by PCR but reported no signs or symptoms. No infections with *A. phagocytophilum* or *Rickettsia* spp. were documented. No infections with *C. burnettii* or *Babesia* spp. were found by PCR; however, serologic testing was not used for these infections.

### Risk for Infection with *B. burgdorferi* s.l.

We compared the demographic and other variables between the participants with *Borrelia* infection and noninfected participants (Table 2). In a multivariate model, the tick engorgement levels (OR 9.52) and confirmation of *B*. *burgdorferi* s.l. in ticks (OR 4.39) showed a major increase in the risk for infection (Table 3).

**Table 2:** Comparison of persons infected and not infected with *Borrelia burgdorferi* sensu lato after tick bite, Austria, 2015–2018*

Variable	Not infected, n = 457			Infected, n = 25		p value
	No. or mean ± SD	Median, % (IQR)		No. or mean ± SD	Median, % (IQR)	
Sex						
M	214	46.8		12	48.0	1.000
F	243	53.2		13	52.0	NA
Age, y	48.7 ± 14.5	48.5 (36.8–59.1)		52.4 ± 14.0	54.0 (42.9–58.6)	0.216
Use of repellent	17	3.7		2	8.0	0.258
No. ticks	1.3 ± 1.2	1.0 (1.0–1.0)		2.4 ± 3.8	1.0 (1.0–2.0)	<0.001
Time, tick bite to blood test, d†	4.3 ± 4.0	4.0 (2.0–6.0)		3.9 ± 2.1	3.0 (2.0–5.0)	0.645
Duration of tick attachment, d	1.0 ± 2.9	1.0 (0.0–2.0)		1.2 ± 1.2	1.0 (0.0–2.0)	0.668
Tick location						
Left leg	119	26.0		15	60.0	<0.001
Right leg	130	28.4		13	52.0	0.022
Left arm	53	11.6		6	24.0	0.106
Right arm	55	12.0		4	16.0	0.530
Head/neck	21	4.6		1	4.0	1.000
Abdomen/chest	71	15.5		4	16.0	1.000
Genital/pelvic area	111	24.3		5	20.0	0.811
Back	46	10.1		4	16.0	0.314
Antimicrobial drug‡	30	6.6		0	0.0	0.39
PCR positive	62	13.6		11	44.0	<0.001
IgG§	57	12.5		6	24.0	0.08
IgM§	30	6.6		2	8.0	0.58
IgG and IgM§	23	5.0		2	8.0	0.37
History of erythema migrans	84	18.0		8	32.0	0.15
Tick engorgement						
None	180	39.5		4	16.0	<0.001
Slightly/partially	219	48.0		10	40.0	NA
Fully	57	12.5		11	44.0	NA

*IQR, interquartile range; NA, not applicable.  †Time between tick bite and first blood test.  ‡Received within 4 weeks before tick bite.  §Presence of *Borrelia*-specific antibodies at the first visit.

**Table 3:** Multiple logistic regression analysis for assessing risk for infection with *Borrelia burgdorferi* sensu lato after tick bite, Austria, 2015–2018*

Parameter	p value	OR (95% CI)
Sex	0.818	0.90 (0.38–2.15)
Age	0.662	1.01 (0.98–1.04)
No. ticks	0.048	1.18 (1.00–1.39)
Tick PCR positive for *B. burgorferi*	0.001	4.39 (1.78–10.84)
Tick engorgement		
Fully	<0.001	9.52 (2.79–32.45)
Slightly/partially	0.229	2.09 (0.63–6.98)
Not engorged	NA	1 (NA)

*NA, not applicable; OR, odds ratio.

We also compared the differences in the distribution of ticks co-infected with multiple pathogens that had bitten participants with and without *Borrelia* infection. Of 37 ticks detached by 25 *Borrelia*-infected participants, 4 (10.8%) harbored >1 pathogen, whereas among 1,242 ticks from the noninfected group, 56 (4.5%) carried multiple pathogens (p = 0.07).

## Discussion

We investigated 482 persons bitten by ticks for the occurrence of bacterial tickborne infections and *Babesia* spp. We demonstrated a high incidence of infections with the emerging pathogen, *Candidatus* N. mikurensis. Furthermore, our data clearly show that *R. helvetica*, though highly abundant in ticks in Austria, does not pose a risk for human health. We also conducted a detailed risk analysis for contracting Lyme borreliosis by analyzing numerous demographic and clinical parameters. This knowledge is needed for further research on the efficacy of specific interventions for preventing Lyme borreliosis, such as local or systemic antimicrobial drug prophylaxis after tick bite (*18*).

The risk for contracting *Borrelia* infection was 5.1%, which is consistent with published data for the Netherlands and Sweden (*14*,*15*), despite a different frequency of *B. burgdorferi* s.l. in ticks (15.2%) compared with previous reports (26% and 29.3%). This finding might be explained by the fact that more larvae were removed during the current study. *I. ricinus* larvae do not harbor *B. burgdorferi* s.l. because of lack of transovarial transmission of this pathogen. An investigation of ticks collected from vegetation throughout Austria showed that 25% of ticks were positive for *Borrelia* spp. (*2*), but no larvae were analyzed. Because male adult *I. ricinus* ticks rarely feed on humans, only 9 of 112 adult ticks detached by study participants were male.

The presence of *Borrelia* in ticks and the level of tick engorgement were the major predictors of infection. However, we did not find a correlation between infection and the time of attachment reported by the participants. Clinical trials on the relationship between infection risk and duration of tick feeding are scarce, and results are contradictory (*14*,*15*). Inconsistency might be attributed to the fact that self-assessment of the duration of tick attachment might be imprecise. We assume that if more granular time intervals (e.g., in hours instead of days) had been applied in our study, the results might have been different, particularly for the large group of persons who had removed their ticks within the first 24 hours (≈50% of the ticks in this study). Transmission of *B. burgdorferi* s.l. can occur <24 hours from tick attachment (*14*,*19*). Our study demonstrates that morphologic evaluation of tick engorgement is more reliable as a predictor for risk of infection. The risk was 10 times higher for fully engorged ticks than for nonengorged ticks. Limited correlation between self-reported duration of tick attachment and level of engorgement has been reported (*20*).

Our data suggest that a history of erythema migrans and presence of antibodies do not avert further *Borrelia* infections. The frequency of participants who had been seropositive at baseline and of those who had previous erythema migrans was higher in the infected group (Table 2). Although not statistically significant, these results suggest greater exposure to ticks.

Among other tickborne pathogens, *Candidatus* N. mikurensis was the most frequent agent identified in blood containing EDTA, and 2% of the participants had an asymptomatic infection with this emerging pathogen. Infection with *Candidatus* N. mikurensis can have a severe clinical picture. Life-threatening complications can occur not only for immunocompromised patients but also for immunocompetent patients (*21*,*22*). The pathogen was detected in blood samples of patients who had erythema migrans–like rashes in Norway; a total of 70 symptomatic patients were tested, and the pathogen was found in 10% of the patients (*23*). Asymptomatic infections are rare and they have been reported in healthy foresters from Poland (*24*), but no prospective data on the risk for acquiring the infection after tick bites are available. For 3 persons, we detected the pathogen in 2 consecutive samples. In 1 of these persons, the first positive sampling occurred during October, and the follow-up was performed 86 days later in January. Because no tick bites were documented in this study during the months of December–February, this finding suggests a long persistence of the pathogen in the blood in the absence of symptoms. However, there are no comparable reports on the persistence of *Candidatus* N. mikurensis in a human host.

*B. miyamotoi* is transmitted uniquely by *Ixodes* ticks and is an emerging pathogen causing febrile illness and meningitis in immunocompromised patients (*25**,**26*). With a prevalence of 2% in ticks, we expected a low incidence of infections in humans. We detected this spirochete in a healthy 79-year-old man. The incidence for infections with *A. phagocytophilum* was low, which corresponds to observations from Scandinavian countries (*27*). We did not document any case despite a relatively high level of background seroprevalence at study inclusion (8%). Severe cases of human granulocytic anaplasmosis sporadically occur in Austria (*28*), and a larger sample size might be necessary to detect such cases.

*Rickettsia* spp. was found in 9.4% of the ticks in our study. However, we did not identify any infections by using serologic or molecular methods. No study participant showed development of clinical signs of rickettsial infection, such as skin eschars or lymphadenopathy. The dominating species in ticks from Austria was *R. helvetica,* and only a few infections with this organism have been reported worldwide, suggesting its low pathogenicity (*16**,**29*).

We identified *Candidatus* R. mendelii in 4 ticks. This novel organism was initially identified in the Czech Republic during 2016 (*30*). Extensive data on its geographic distribution are missing. We also detected a new *Rickettsia* sp. of the spotted fever group in a tick from Tyrol, Austria (*17*).

We did not exclude patients who had received previous antimicrobial drug treatment. A total of 23 of these patients received antimicrobial drugs that were active against tickborne pathogens starting 4 weeks before enrollment. Six participants were receiving antimicrobial drugs at study inclusion, and the time point for antimicrobial drug treatment was not known exactly for 7 participants. Two participants were receiving immunosuppressive treatment. For persons with multiple tick bites in the noninfected group, we randomly selected 1 tick for risk analysis because it would otherwise have been difficult to calculate a regression model. Finally, for some pathogens, we used PCR only to identify infections without additional serologic testing, including that for *Babesia* spp. and *B. miyamotoi*. Because of a low prevalence of these pathogens in ticks, it is unlikely that we would have found a substantial amount of infections by using serologic methods.
